# Transcutaneous auricular vagus nerve stimulation inhibits mental stress‐induced cortisol release—Potential implications for inflammatory conditions

**DOI:** 10.14814/phy2.70251

**Published:** 2025-02-12

**Authors:** Ely Cuberos Paredes, Domenica Goyes, Sadie Mak, Raffi Yardimian, Nickolas Ortiz, Ayana McLaren, Harald M. Stauss

**Affiliations:** ^1^ Department of Biomedical Sciences Burrell College of Osteopathic Medicine Las Cruces New Mexico USA

**Keywords:** anti‐inflammatory reflex, glucocorticoid resistance, hypothalamus‐pituitary–adrenal axis, psoriasis

## Abstract

Elevated glucocorticoid levels with reduced glucocorticoid responsiveness have been reported in chronic inflammatory conditions. Activation of neurons in the nucleus of the solitary tract by transcutaneous auricular vagus nerve stimulation (taVNS) may activate inhibitory pathways projecting to the hypothalamic paraventricular nucleus (PVN), thus inhibiting corticotropin‐releasing hormone (CRH) release and improving glucocorticoid dysfunction in chronic inflammatory conditions. Healthy adults (*n* = 12) participated in experimental (taVNS) and control (sham‐taVNS) sessions at least 4 days apart. A 30‐min baseline recording was followed by 30 min of taVNS or sham‐taVNS and 40 min of recovery. Ten minutes into taVNS or sham‐taVNS, a mental arithmetic stress test (MAST) was conducted for 15 min. The MAST increased heart rate, low frequency (LF) heart rate variability (HRV), and the LF to high frequency ratio of HRV, confirming sympathetic activation. Salivary cortisol levels during the MAST were lower during taVNS (49.5 ± 48.0% from baseline; mean ± SD) compared to sham‐taVNS (106.0 ± 81.1% from baseline; mean ± SD; *p* < 0.05). In a psoriasis patient, daily taVNS for 3 months reduced diurnal salivary cortisol levels from 58.2 ± 35.2 (ng/mL)*h (mean ± SD) to 34.9 ± 13.8 (ng/mL)*h (mean ± SD). While it is possible that taVNS inhibited CRH‐releasing neurons in the PVN, our study design did not allow to confirm this potential mechanism.

## Graphical abstract

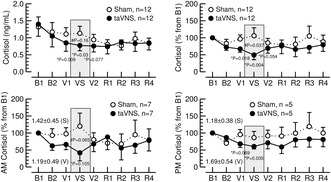

## 1 INTRODUCTION

The negative impact of stress on the human body has already been recognized by Galen of Pergamon (129–216 AD), who stated in his treatise on the preservation of health (De Sanitate Tuenda) that “rage, weeping, anger, distress and excessive worry, and poor sleep arising from these, provoke fevers, and become the starting point of major diseases”. Moving forward two millennia, life stress has been implicated with many chronic conditions, including neoplastic (Eskelinen & Ollonen, 2010; Pereira et al., 2010), cardiovascular (Bautista et al., 2019; Low et al., 2009; Rosmond & Bjorntorp, 2000), and inflammatory diseases (Kozora et al., 2005; Rimón & Laakso, 1985). The major neural/endocrine pathways activated by life stressors are the sympathetic nervous system and the hypothalamus‐pituitary–adrenal (HPA) axis. With regard to the HPA axis, a population‐based study in 869 adults found that a large area under the curve (AUC) of diurnal salivary cortisol concentrations determined at 6 time points between awakening and bedtime are associated with high interleukin‐6 (IL‐6) plasma levels (DeSantis et al., 2012). Due to the known anti‐inflammatory effects of glucocorticoids, this finding may be surprising. However, it has been suggested that chronic exposure to life stress may result in dysregulation of the HPA axis with elevated cumulative diurnal cortisol levels. This may result in downregulation of glucocorticoid receptors on immune cells, which would impair the ability of endogenous glucocorticoids to inhibit immune cell function, resulting in chronic inflammation (DeSantis et al., 2012; McEwen et al., 1997; Miller et al., 2002). This idea is in line with experiments demonstrating reduced efficacy of dexamethasone to inhibit lipopolysaccharide‐stimulated production of interleukin 1β (IL‐1β), IL‐6, and tumor necrosis factor‐α (TNF‐α) in blood samples from subjects pretreated for 2 weeks with dexamethasone (Lekander et al., 2009). These considerations give rise to the intriguing possibility that lowering cortisol levels in patients with chronic inflammatory diseases may resolve HPA dysfunction by restoring glucocorticoid receptor density on immune cells which may ultimately improve chronic inflammatory conditions. Such an approach may be particularly effective in patients exposed to high levels of life stress associated with elevated cumulative diurnal cortisol levels.

The vagus nerve is in a key position to modulate endogenous cortisol levels through its afferent pathway. As part of the cholinergic anti‐inflammatory reflex (Tracey, 2002) afferent vagal nerve fibers sense inflammatory insults throughout the body and signal to the nucleus of the solitary tract (NTS). Viral tracing studies identified direct pathways between the NTS and inhibitory GABA‐ergic neurons surrounding the PVN (Affleck et al., 2012), the major site of corticotropin‐releasing hormone (CRH) production. These pathways may provide a neuroanatomical mechanism by which afferent vagal nerve stimulation (VNS) may inhibit the HPA axis. Indeed, glucocorticoid receptor stimulation in the NTS has been demonstrated to attenuate the peripheral corticosterone response to restraint stress in rats (Ghosal et al., 2014). On the other hand, projections from the NTS can activate norepinephrinergic neurons in the locus coeruleus (LC) that can activate the HPA axis (Dunn et al., 2004). With this regard, Warren et al. (Warren et al., 2019) reported a decrease in salivary cortisol levels during a 75‐min oddball experiment in study participants that received sham‐transcutaneous auricular VNS (taVNS), while cortisol levels remained constant in participants that received active taVNS. Similar salivary cortisol responses to sham‐taVNS and active taVNS were found by D'Agostini et al. in a study on reversal learning (D'Agostini et al., 2021). These findings suggest that taVNS, which mainly activates afferent vagal nerve fibers (Butt et al., 2020; Nomura & Mizuno, 1984) that project to the NTS (Nomura & Mizuno, 1984; Toschi et al., 2023; Yakunina et al., 2017), works against the normal diurnal tendency for salivary cortisol levels to decrease. The prevailing HPA axis activity may be a key factor determining whether afferent VNS inhibits or activates HPA axis activity. The studies by Warren et al. (2019) and D'Agostini et al. (2021) focused on novelty processing and learning, respectively. Thus, the experimental conditions were not necessarily associated with elevated HPA axis activity. Under these conditions, afferent VNS prevented the normal diurnal decrease in cortisol levels. In contrast, afferent VNS seems to inhibit HPA axis activity under experimental or clinical conditions associated with elevated HPA axis activity, such as restraint stress in rats (Ghosal et al., 2014), CRH application in rodents (Chen et al., 2021), CRH challenge test in patients with major depressive disorder (O'Keane et al., 2005), or in a rodent model of chronic mild stress (Liu et al., 2013).

To further investigate the idea that taVNS inhibits the HPA axis response to stress, we hypothesized that afferent VNS through transcutaneous auricular VNS (taVNS) inhibits the cortisol response to mental arithmetic stress in generally healthy adults. Testing this hypothesis will potentially add to the mounting evidence that afferent VNS suppresses the HPA axis response to stress (Chen et al., 2021; Liu et al., 2013; O'Keane et al., 2005). In addition, the results of this study may potentially provide a mechanistic rationale for the use of afferent VNS in chronic inflammatory conditions. With this regard, we also present a case report of a psoriasis patient who participated in our ongoing clinical trial (ClinicalTrials.gov ID NCT05243303) on the effects of taVNS in plaque psoriasis. This patient provided saliva samples collected at six time points throughout the day before and during a 3‐month taVNS trial. These samples allowed us to investigate the effect of chronic taVNS on salivary cortisol concentrations in a patient with a chronic inflammatory condition.

## 2 METHODS

### 2.1 Study participants

The study was approved by the Burrell College Institutional Review Board (BURRELL IRB 0112_2023), and all participants (*n* = 12, 6 female, 6 male) provided informed written consent. Study participants were required to be at least 18 years of age. Exclusion criteria included pregnancy, any acute or chronic diseases, or medications that may interfere with the outcome of the study or that may increase the risk associated with the procedures of the study.

### 2.2 Experimental protocol

**FIGURE 1:**
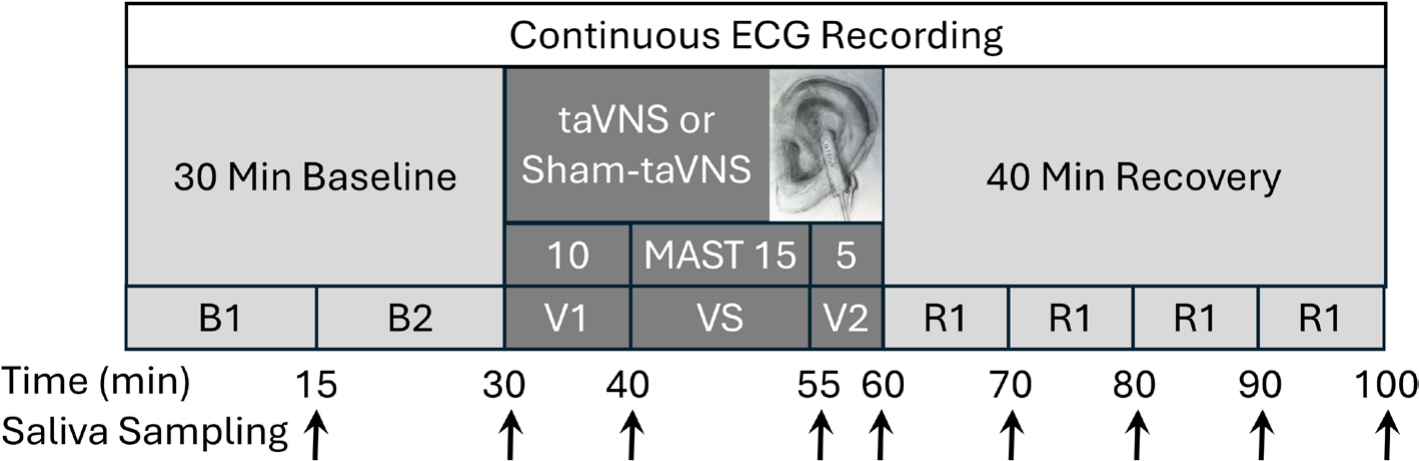
Study participants reported for two study sessions at least 1 week apart. Throughout the study sessions the electrocardiogram (ECG) was recorded continuously. Each study session consisted of a 30‐min baseline recording, followed by 30 min of either transcutaneous auricular vagus nerve stimulation (taVNS, experimental study day) or sham‐taVNS (control study day). Electrode placement is shown in the inset. The order of the experimental versus control study day was randomized. Ten minutes into the taVNS or sham taVNS intervention, a mental arithmetic stress test (MAST) was conducted for 15 min. The 30 min of taVNS or sham‐taVNS were followed by a 40‐min recovery period. B1, B2, V1, VS, V2, R1, R2, R3, and R4 represent segments within the experimental protocol that were used for heart rate variability analysis. Throughout the experimental protocol saliva samples were collected at 15, 30, 40, 55, 60, 70, 80, 90, and 100 min (black arrows on bottom of figure).

All experiments were completed within the month of June of 2023 at the clinic rooms within the main building of Burrell College of Osteopathic Medicine in Las Cruces, NM. Thus, potential seasonal effects can be excluded. The clinic rooms were air‐conditioned with the temperature set at 72.5 F (22.5°C). Study participants were instructed to abstain from caffeinated beverages on the day of the experiments but otherwise were allowed to maintain their normal daily routine. The experimental protocol is illustrated in Figure 1. Each of the 12 participants underwent two study sessions separated by a minimum of 4 days. In 7 participants both sessions were conducted in the morning (AM) and in 5 participants both sessions were conducted in the afternoon (PM). The two study sessions differed in whether transcutaneous auricular vagus nerve stimulation (taVNS, experimental condition) or a sham‐taVNS intervention (control condition) was conducted. On the first study day, participants provided written informed consent and answered an online questionnaire verifying the absence of exclusion criteria. On each of the two study days, height and weight (Model 1,127,154, Henry Schein Medical, Melville, NY or Model 500KL, Pelstar LLC/Health‐O‐Meter, McCook, IL) as well as blood pressure and heart rate (Model BP786N, OMRON, Lake Forest, IL) were measured before the start of the experimental protocol. Throughout the experimental protocol, participants were in supine position, and the electrocardiogram (ECG) was recorded continuously. After a 30‐min baseline recording either taVNS (experimental study day) or sham‐taVNS (control study day) was applied for another 30 min. The order of the experimental versus control study day was randomized, and study participants were blinded regarding the sham‐taVNS or taVNS intervention. Ten minutes into the taVNS or sham‐taVNS intervention, a mental arithmetic stress test (MAST) was conducted for 15 min. The 30‐min taVNS or sham‐taVNS intervention was followed by a 40‐min recovery period. Saliva samples were collected at 15, 30, 40, 55, 60, 70, 80, 90, and 100 min into the experimental protocol (Figure 1). Thus, two saliva samples were collected before initiating taVNS or sham‐taVNS, one sample was obtained immediately before and after the MAST, and five samples were collected throughout the recovery period.

### 2.3 Transcutaneous auricular vagus nerve stimulation (taVNS)

For taVNS, a transcutaneous electrical nerve stimulator (EMS 7500; Current Solutions, LLC, Austin, TX) was used. The device is registered with the FDA under 510(k) premarket notification K080661. A bipolar clip electrode (see inset in Figure 1) was applied to the cymba conchae of the left ear that is innervated by the auricular branch of the vagus nerve (Arnold's nerve) (He et al., 2012). Prior to attaching the electrode, conductive electrode gel (Spectra 360, Parker Laboratories, Inc., Fairfield, NJ) was applied. The cathode of the bipolar electrode was placed at the cavum of the concha, and the anode was placed at the opposing site of the back of the auricle that is also innervated by the vagus nerve. The stimulation parameters were 10 Hz stimulation frequency, 300 μs pulse width, and duty cycle of constant (100%) on. The stimulation current was determined individually for each subject by slowly increasing the stimulation current until the subjects felt a mild tingling sensation at the site of the electrode. Then the current was gradually reduced until the tingling sensation disappeared or was just barely felt. Consistently, the resulting stimulation current was between 2.0 and 2.5 mA. The stimulation was applied for 30 min (Figure 1). No adverse events were noticed during the study.

### 2.4 Sham‐taVNS

The sham‐taVNS intervention was conducted in the same manner as the taVNS intervention, with the exception that the electrode cable was not connected to the EMS 7500 device. Thus, no current was applied to the ear. To reinforce blinding, investigators pretended to slowly increase the stimulation current as for the taVNS intervention, while repeatedly asking participants if they feel a tingling sensation. Importantly, all participants reported feeling this sensation during the sham‐taVNS application, despite no current being delivered to the electrode. It is possible that the pressure sensation of the clip electrode resulted in this perception. Thus, it is reasonable to believe that study participants were truly blinded regarding the experimental vs. control condition. The sham‐taVNS intervention was applied for 30 min (Figure 1).

### 2.5 Mental arithmetic stress test (MAST)

For the MAST, participants were instructed to count aloud backwards from a 3‐digit number (e.g., 517) by odd decrements (e.g., 7). Investigators interrupted the counting, regardless of accuracy, to prompt subjects to start over. The MAST was applied for 15 min.

### 2.6 Heart rate variability (HRV) analysis

HRV analysis is based on 24 ECG recordings obtained on two study days in 12 study participants and was conducted in the time and frequency domain. ECGs were recorded at a sampling rate of 500 Hz, using the WinAD data acquisition module of the freely available HemoLab software (Stauss, 2024). Using the Analyzer module of the HemoLab software, the heart rate (HR) and RR‐intervals were extracted from the original ECG recordings on a beat‐by‐beat basis. These HR and RR‐interval time series were split into nine individual time series for the first and second 15 min of the baseline recording (B1, B2 in Figure 1), the first 10 min of the taVNS/sham‐taVNS intervention before the start of the MAST (V1 in Figure 1), the MAST (VS in Figure 1), the final 5 min of the taVNS/sham‐taVNS intervention (V2 in Figure 1), and four 10 min segments during the recovery recording (R1, R2, R3, and R4, in Figure 1). Artifacts (e.g., movement artifacts, PVCs, less than 1% of all heart beats) were replaced by interpolations based on preceding and trailing values. Using the Batch Processor module of the HemoLab software, these beat‐by‐beat HR and RR‐interval time series were spline‐interpolated to an equidistant sampling rate of 15 Hz. These 15 Hz time series were used for time and frequency domain analysis. For frequency domain analysis, the FFT algorithm and 50% overlapping segments of 2048 data points (136 s) were used. Absolute low frequency (LF) spectral power of heart rate (in bpm^2^) was determined in the frequency range of 0.04–0.15 Hz (LF_HR_) and high frequency (HF) power of RR‐intervals (in ms^2^) was determined in the frequency range of 0.15–0.40 Hz (HF_RR_) as suggested by the Task Force of The European Society of Cardiology and The North American Society of Pacing and Electrophysiology (Task Force of the European Society of Cardiology and the North American Society of Pacing and Electrophysiology, 1996). LF power was determined from heart rate time series. and HF power was determined from RR‐interval time series, because it has been demonstrated that LF HRV best predicts sympathetic tone when calculated from heart rate time series, while HF HRV best predicts parasympathetic tone when calculated from RR‐interval time series (Kania et al., 2024). Consequently, the LF to HF ratio, a measure of “autonomic balance” was calculated as the ratio of the LF power from heart rate time series to the HF power from RR‐interval time series (LF_HR_/HF_RR_). For time domain analysis, 50% overlapping segments of 4‐min long beat‐by‐beat RR‐interval time series were used to calculate the standard deviation of all normal to normal (NN) intervals (SDNN) and the square root of the mean of the sum of the squares of differences between adjacent NN intervals (RMSSD) according to the Task Force of The European Society of Cardiology and The North American Society of Pacing and Electrophysiology (Task Force of the European Society of Cardiology and the North American Society of Pacing and Electrophysiology, 1996).

### 2.7 Salivary cortisol concentration

Saliva was collected using a Saliva Collection Aid (Salimetrics, State College, PA) at 9 time points throughout the experimental protocol (Figure 1). Study participants were not instructed to brush their teeth immediately prior to the experiments to avoid potential blood contamination of saliva samples that may occur in response to tooth brushing in study participants that may be affected by gingivitis. Study participants were instructed to produce about 1 mL of saliva. Saliva flow rate was determined as the volume of saliva produced (in μL) divided by the time (in seconds) needed to produce the saliva sample. Samples were stored at −80°C until analysis. Saliva cortisol concentrations were determined in quadruplicate using a commercially available enzyme‐linked immunosorbent assay (ELISA, K003‐H1, Arbor Assays, Ann Arbor, MI) following the manufacturer's instructions.

### 2.8 Statistics

Statistical analysis was performed using the R Statistical Software (R Core Team, 2020). Data are presented as means ± standard deviation (SD) unless otherwise noted. Statistical analysis of heart rate, HRV, and salivary cortisol data was performed by 2‐way Analysis of Variance (ANOVA) for two repeated measures (taVNS versus sham‐taVNS condition and time points throughout the experimental protocol). Post‐hoc *t*‐tests with Bonferroni correction were performed to compare time interval B2 (15–30 min) for HR and HRV or time point B1 (15 min) for salivary cortisol concentrations with all subsequent time points (see Figure 1). Post‐hoc paired *t*‐tests with Bonferroni correction were performed to compare the two experimental conditions (sham‐taVNS vs. taVNS) for each time point. The Shapiro test was used to test for normality of the data. In case of non‐normality, data were transformed using the “Yeo‐Johnson” (LF_HR_, SDNN, ΔLF_HR_), “Box Cox” (HR, HF_RR_), “arcsinh” (LF_HR_/HF_RR_, RMSSD, ΔLF_HR_/HF_RR_, ΔHF_RR_), or “ordered quantile normalizing” (absolute cortisol, percent cortisol from time point B1) transformation as determined by the bestNormalize function of the bestNormalize R package. No transformations were necessary for ΔHR, ΔSDNN, ΔRMSSD because these data were normally distributed. Statistical significance was assumed for *p* < 0.05, and trends were considered for *p* ≤ 0.15.

### 2.9 Case report

We report from a 56‐year‐old female patient with mild plaque psoriasis (Psoriasis Area and Severity Index of 1.5) who participated in our ongoing clinical trial on the effects of taVNS on plaque psoriasis. The study is registered with ClinicalTrials.gov (NCT05243303) and is approved by the Burrell College Institutional Review Board (BURRELL IRB 0090_2021). In this study, patients self‐administer taVNS (same device and electrode as described above, 10 Hz frequency, 300 μs pulse width, 2.0–2.5 mA current, 100% on duty cycle) every day for 3 months for 30 min before bedtime. The patient collected saliva samples before (days −9, −8, −2, and −1) and during (days 11, 12, 35, 66, 90, and 91) the taVNS protocol. On each saliva collection day, the patient collected six saliva samples (6:00 am, 9:00 am, noon, 3:00 pm, 6:00 pm, and 9:00 pm). Salivary cortisol concentrations were determined using a commercially available enzyme‐linked immunosorbent assay (ELISA, K003‐H1, Arbor Assays, Ann Arbor, MI) following the manufacturer's instructions. For each time of the day, the salivary cortisol concentrations were averaged for the 4 days before initiation of taVNS (baseline), for days 11 and 12 of the taVNS protocol (early taVNS), and for days 35, 66, 90, and 91 of the taVNS protocol (chronic taVNS). We also calculated the cumulative salivary cortisol concentration as the area under the curve (AUC) for the 15 h from 6:00 am to 9:00 pm for the baseline days before taVNS and for the early and chronic taVNS time points. We refrained from statistical analyses because the data are based on a single subject.

## 3 RESULTS

### 3.1 Characteristics of study participants

A total number of 12 individuals (6 female, 6 male) participated in the study. Average age was 26.2 ± 4.2 years; body mass index (BMI) was 25.3 ± 3.7 kg/m^2^; resting systolic blood pressure (BP) was 119 ± 12 mmHg; resting diastolic BP was 79 ± 7 mmHg; and resting heart rate (HR) was 75 ± 8 bpm (baseline values from both study days averaged, means±SD). Four participants took dietary supplements (minerals, vitamins, and/or probiotics), one participant took trazodone as needed for insomnia and another participant used an albuterol inhaler as needed and an oral contraceptive. One participant reported chronic sinusitis and a degenerative vertebral disc condition, and another participant reported to be affected by attention‐deficit/hyperactivity disorder but was not taking any medication for this condition. No other medications or medical conditions were reported.

### 3.2 Autonomic responses to the MAST

**FIGURE 2:**
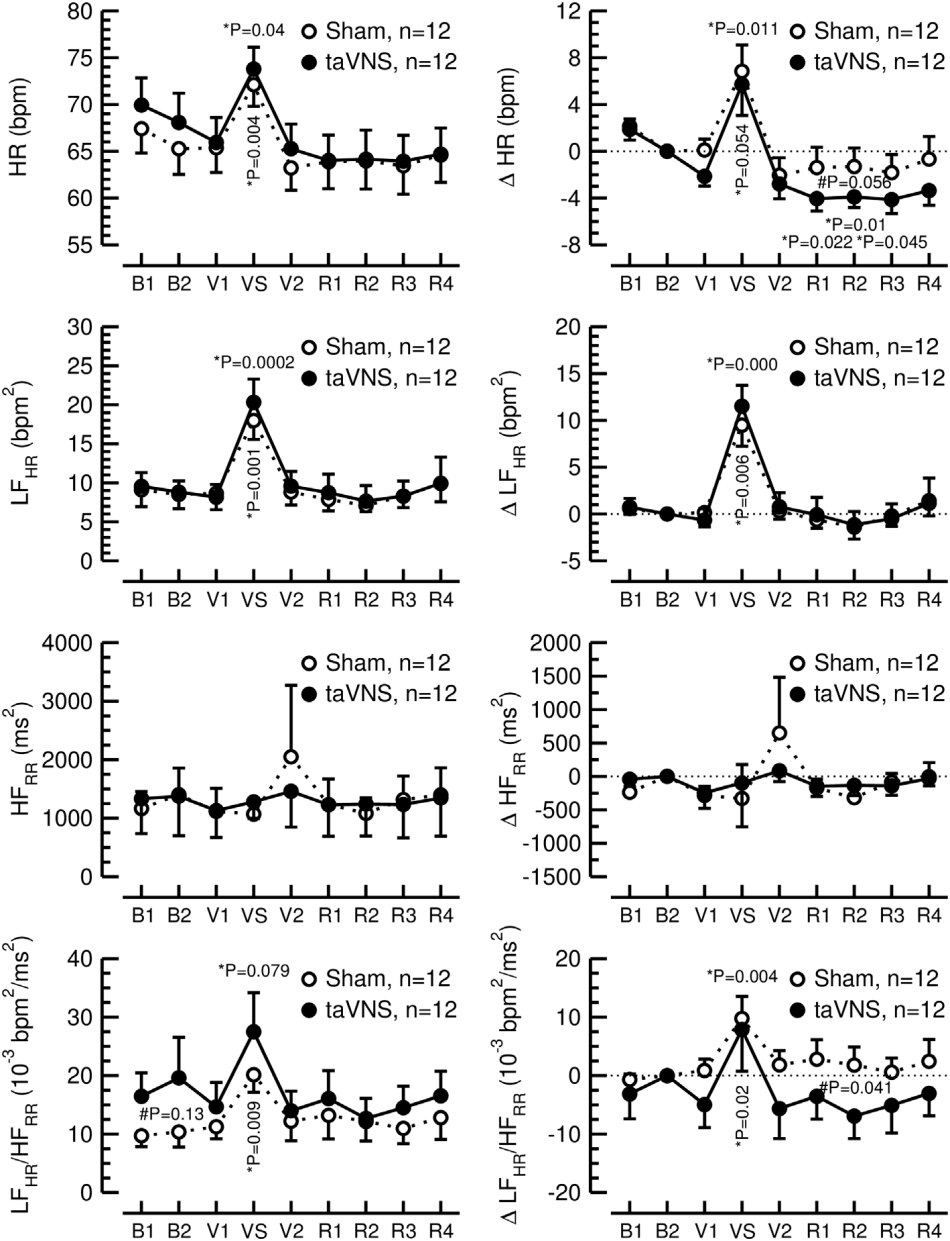
Heart rate (HR, top row) and heart rate variability expressed as low frequency heart rate variability (LF_HR_, 2nd row), high frequency RR‐interval variability (HF_RR_, 3rd row), and LF_HR_ to HF_RR_ ratio (LF_HR_/HF_RR_, bottom row). The left column shows absolute data, the right column shows changes (Δ) from time interval B2 (15–30 min, see Figure 1). Error bars represent standard errors of the mean. #: Sham‐taVNS vs. taVNS; *: Respective time interval vs. time interval B2.

Mental arithmetic stress (time point VS) significantly raised heart rate (time effect in 2‐way ANOVA: DFn = 2.09, DFd = 22.97, *F* = 10.13, *p* = 0.001; Figure 2, top left), LF_HR_ (time effect in 2‐way ANOVA: DFn = 8, DFd = 88, *F* = 12.02, *p* < 0.001; Figure 2, 2nd row left), and LF_HR_/HF_RR_ ratio (time effect in 2‐way ANOVA: DFn = 2.08, DFd = 22.88, *F* = 4.85, *p* = 0.017, Figure 2, bottom row left), confirming that the MAST increased sympathetic activity. While there was a trend towards an increase in SDNN in response to the MAST (time effect in 2‐way ANOVA: DFn = 8, DFd = 88, *F* = 1.91, *p* = 0.068), there were no significant effects of the MAST on RMSSD (time effect in 2‐way ANOVA: DFn = 2.19, DFd = 24.13, *F* = 0.53, *p* = 0.611) or HF_RR_ (time effect in 2‐way ANOVA: DFn = 1.85, DFd = 20.35, *F* = 0.41, *p* = 0.655, Figure 2, 3rd row left), suggesting that the MAST had no major effects on parasympathetic modulation of sinus node automaticity.

### 3.3 Salivary cortisol responses to the MAST

**FIGURE 3:**
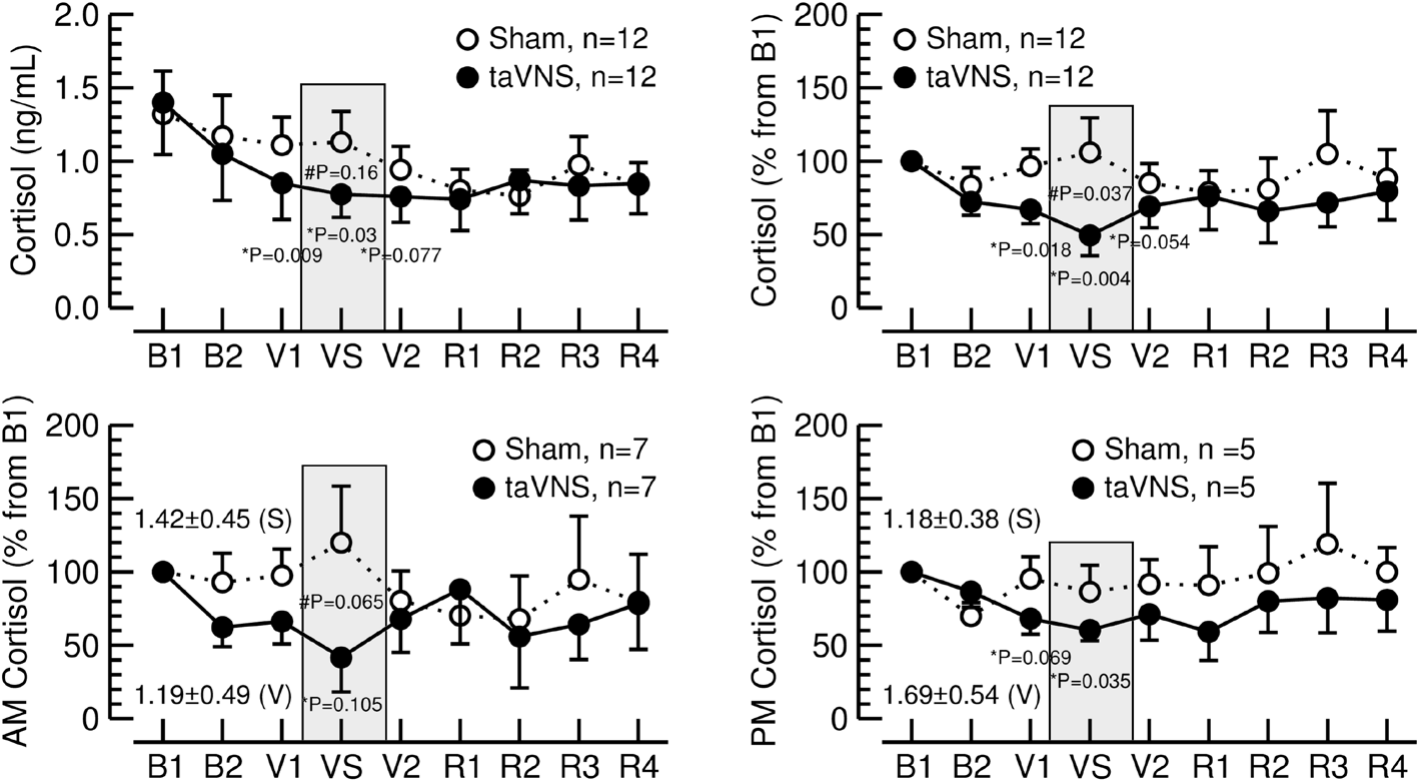
Top row: Salivary cortisol concentrations in absolute units (ng/mL, left) and in percent from time point B1 (15 min, right). The data from all 12 study participants are included. Error bars represent standard errors of the mean. Bottom row: In 7 study participants, the experiments were performed in the morning (AM) and in 5 study participants, the experiments were performed in the afternoon (PM). For individual study participants, the sham‐taVNS and taVNS experiments were always performed at the same time of the day (either AM or PM). Salivary cortisol concentrations expressed as percent from time point B1 (15 min) are shown for the experiments performed in the AM (left) and PM (right). The absolute cortisol concentrations at time point B1 (in ng/mL) are provided for the sham‐taVNS (S) and taVNS (V) conditions. #: Sham‐taVNS versus taVNS; *: Respective time interval versus time interval B1. Error bars represent standard errors of the mean.

Consistent with the diurnal changes in cortisol levels, there was an overall trend towards a decrease in absolute salivary cortisol levels throughout the 100 min of the experimental protocol (time effect in 2‐way ANOVA: DFn = 2.14, DFd = 23.51, *F* = 2.22, *p* = 0.128, Figure 3, top, left). During the sham‐taVNS condition, this overall trend did not reach statistical significance. Thus, the MAST appears to work against a general tendency for cortisol secretion to decrease over the course of the experimental protocol.

### 3.4 Effect of time of day on salivary cortisol levels

Because of the diurnal changes in cortisol levels, one would expect lower salivary cortisol concentrations in the afternoon than in the morning (Ljubijankić et al., 2008). However, baseline salivary cortisol levels were not significantly different in the 7 study participants who reported to the laboratory in the morning (1.30 ± 0.92 ng/mL at time point B1, data from sham‐taVNS and taVNS protocols pooled, Figure 3 bottom left) compared to the 5 study participants who were studied in the afternoon (1.43 ± 1.02 ng/mL at time point B1, data from sham‐taVNS and taVNS protocols pooled, Figure 3 bottom right).

### 3.5 Potential effects of saliva flow rates

**FIGURE 4:**
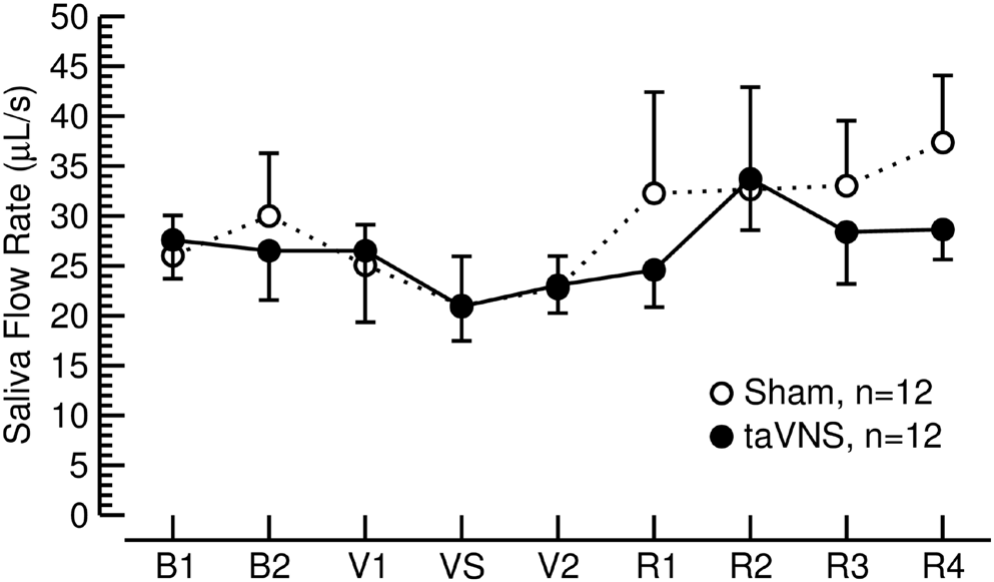
Saliva flow rate did not change significantly throughout the sham‐taVNS and taVNS protocols. Error bars represent standard errors of the mean.

Because high saliva flow rates may result in dilution of cortisol in saliva samples, we determined saliva flow rate. As shown in Figure 4, saliva flow rate did not differ between the sham‐taVNS and taVNS condition and did not change significantly throughout the experimental protocol. Thus, potential changes in saliva flow rate did not confound the saliva cortisol concentrations.

### 3.6 Effect of taVNS on autonomic responses to the MAST

When expressed as differences between the second baseline values (time point B2) and the values during the recovery period (time points R1 to R4), ΔHR (group effect in 2‐way ANOVA: DFn = 1, DFd = 11, *F* = 3.21, *p* = 0.101) and ΔLF_HR_/HF_RR_ ratio (group effect in 2‐way ANOVA: DFn = 1, DFd = 11, *F* = 2.37, *p* = 0.152) tended to decrease to lower levels in the taVNS trial (R2: ΔHR: 3.9 ± 3.1 bpm; ΔLF_HR_/HF_RR_: −6.9 ± 13.3 bpm^2^/ms^2^) compared to the sham‐taVNS trial (R2: ΔHR: −1.3 ± 5.5 bpm, post‐hoc pairwise *t*‐tests: DF = 11, *t* = 2.14, *p* = 0.056, Figure 2 [top right]; ΔLF_HR_/HF_RR_: +1.8 ± 10.7 bpm^2^/ms^2^, post‐hoc pairwise *t*‐tests: DF = 11, *t* = 2.32, *p* = 0.041, Figure 2 [bottom right]). These findings suggest that taVNS either reduced sympathetic activation and/or reduced parasympathetic withdrawal in response to mental arithmetic stress. No group effects were detected for ΔLF_HR_ (group effect in 2‐way ANOVA: DFn = 1, DFd = 11, *F* = 0.05, *p* = 0.83), ΔHF_RR_ (group effect in 2‐way ANOVA: DFn = 1, DFd = 11, *F* = 0.009, *p* = 0.928, Figure 2 [3rd row right]), ΔSDNN (group effect in 2‐way ANOVA: DFn = 1, DFd = 11, *F* = 0.32, *p* = 0.584), or ΔRMSSD (group effect in 2‐way ANOVA: DFn = 1, DFd = 11, *F* = 2.08, *p* = 0.178).

### 3.7 Effect of taVNS on salivary cortisol responses to the MAST

During the intervention (time points V1, VS, V2), salivary cortisol levels expressed in percent from baseline (time point B1, Figure 3 [top right]) were lower during the taVNS trial compared to the sham‐taVNS trial (group effect in 2‐way ANOVA: DFn = 1, DFd = 11, *F* = 4.17, *p* = 0.066). Pairwise post‐hoc t‐tests showed that this group difference was significant during the MAST (time point VS: DF = 11, *t* = 2.37, *p* = 0.037). This effect of taVNS was confirmed for the morning experiments (DF = 6, *t* = 2.26, *p* = 0.065, *n* = 7, Figure 4, bottom left) but not for the afternoon experiments (DF = 4, *t* = 0.94, *p* = 0.399, *n* = 5, Figure 3, bottom right). Furthermore, during the MAST (time point VS), cortisol levels expressed in absolute units (ng/mL, Figure 3 top left) and as percent (Figure 3 top right) were not different from baseline levels (time point B1) during the sham‐taVNS session, but significantly lower than baseline levels during the taVNS session. These results suggest that taVNS inhibits the cortisol response to the MAST.

### 3.8 Case report

**FIGURE 5:**
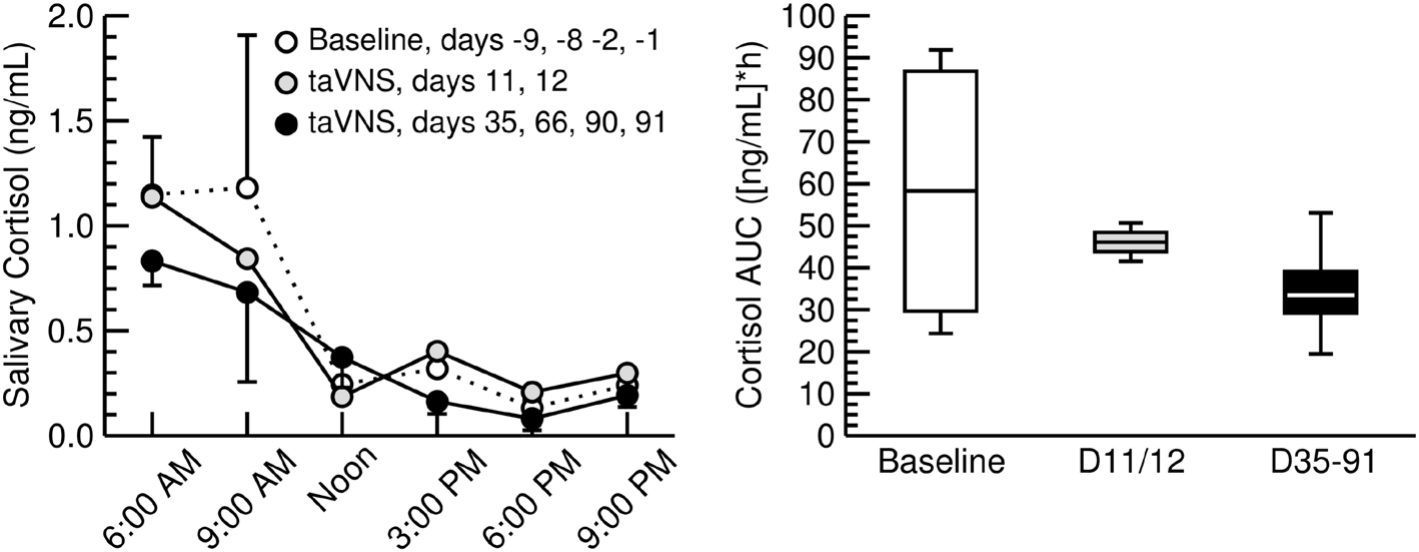
*Left*: Diurnal salivary cortisol concentrations in a single patient with plaque psoriasis before (days −9, −8, −2, and −1, white circles) initiation of a chronic taVNS protocol and during the early (days 11 and 12, gray circles) and chronic (days 35, 66, 90, and 91, black circles) phases of the taVNS protocol. Error bars represent the variability between the respective study days expressed as standard errors of the mean. Right: Cumulative salivary cortisol concentrations in a single patient with plaque psoriasis during the 15 h between 6:00 am and 9:00 pm expressed as the area under the curve (AUC) for the baseline before taVNS (days −9, −8, −2, and −1, white bar) and for the early (days 11 and 12, gray bar) and chronic (days 35, 66, 90, and 91, black bar) phases of the taVNS protocol. Data are shown as Box‐Whisker plots with the “whiskers” representing the upper and lower extreme values, the “box” representing the upper and lower quartiles and the horizontal line representing the median.

Salivary cortisol concentrations demonstrated the expected diurnal pattern with highest values in the morning upon awakening at 6:00 am, followed by a gradual decrease throughout the day (Figure 5, left). The morning cortisol concentrations at 6:00 am and 9:00 am appeared higher before initiation of taVNS (days −9, −8, −2, and −1) compared to the chronic taVNS time points (days 35, 66, 90, and 91). The early taVNS time points (days 11 and 12) seem to fall in between the baseline and chronic taVNS time points. These lower salivary cortisol concentrations during the early and chronic phases of the taVNS protocol compared to the baseline values were confirmed by the cumulative salivary cortisol concentrations expressed as the area under the curve (AUC) of the cortisol concentrations for the 15 hours from 6:00 am to 9:00 pm (Figure 5, right). These AUCs demonstrate 40% lower salivary cortisol levels during the chronic phase of the taVNS protocol (34.9 ± 13.8 (ng/mL)*h) compared to the baseline before taVNS (58.2 ± 35.2 (ng/mL)*h). While definite conclusions cannot be drawn from a single case report, these results appear to confirm the notion that afferent VNS suppresses rather than activates the HPA axis. Patients in our ongoing psoriasis study keep a diary with daily entries of various information that include the time of taVNS and potential adverse effects. So far, none of the enrolled patients, including the patient reported here, have noticed any adverse effects.

## 4 DISCUSSION

The major finding of this study is that taVNS inhibits the cortisol response to a mental arithmetic stress test (MAST) in generally healthy young adults (Figure 3). In line with this finding, chronic taVNS for up to 3 months lowered cumulative diurnal salivary cortisol levels compared to before taVNS in a patient with plaque psoriasis (Figure 5, right). In addition, preliminary unpublished data from our ongoing study demonstrate that chronic taVNS improves the Psoriasis Area and Severity Index (PASI) in patients with plaque psoriasis (9.0 ± 9.4 before taVNS vs. 3.1 ± 2.1 after 3 months of taVNS, *n* = 7, *p* < 0.05, Wilcoxon signed‐rank test). Collectively, these findings provide support for the intriguing possibility that taVNS may suppress an exaggerated HPA axis responsiveness to life stressors, thereby resolving HPA dysfunction and ultimately improving chronic inflammatory disease conditions.

The MAST has been demonstrated to activate the sympathetic nervous system (Lindvall et al., 1991; Saitoh et al., 1995) and to increase serum cortisol concentrations (Jørgensen et al., 1990). To confirm the effectiveness of mental arithmetic stress to increase sympathetic nervous system activity, we recorded the ECG and assessed heart rate and heart rate variability (HRV). The use of HRV to assess “autonomic tone” has been highly debated (Parati et al., 2006; Taylor & Studinger, 2006). However, it has been demonstrated in animal experiments that cardiac sinus node cells are too sluggish to respond to β_1_‐adrenergic receptor stimulation at the respiratory frequency (Stauss et al., 1997). Thus, absolute HF HRV, coinciding with the respiratory frequency, is exclusively modulated by the parasympathetic nervous system, while LF HRV, coinciding with a 10 s periodicity in humans, can respond to both sympathetic and parasympathetic activity. This concept is in line with β_1_‐adrenergic and M_2_‐muscarinic effects on the action potential of sinus node cells (Kania et al., 2024; Monfredi et al., 2014). However, it is important to note that HRV is not a measure of “autonomic tone”, but rather a measure of “autonomic modulation of sinus node function”, because HRV does not only depend on autonomic nervous system activity, but also on the mean level of heart rate (Monfredi et al., 2014; Stauss, 2014) and on cardiac autonomic responsiveness. The effect of the mean level of heart rate can be minimized by calculating LF HRV from heart rate time series and HF HRV from RR‐interval time series (Kania et al., 2024) as has been done in this study. In the current study, the MAST increased heart rate during sham‐taVNS and taVNS (time point VS in Figure 2). In addition, absolute LF_HR_ increased significantly under both experimental conditions, while absolute HF_RR_ was not affected by the MAST. Because absolute HF_RR_ did not change, the increase in LF_HR_ confirms that the MAST in our hands increased sympathetic nervous system activity directed to the heart and potentially to other organs.

Non‐invasive taVNS has been demonstrated by us (Dalgleish et al., 2021; Kania et al., 2021, 2024) and others (Forte et al., 2022) to modulate autonomic nervous system activity, although a recent meta‐analysis questioned such effects (Wolf et al., 2021). In our study, heart rate and the low frequency to high frequency ratio of HRV were lower following the intervention (time points R1‐R4 in Figure 2) compared to baseline (time point B2 in Figure 2) during the taVNS but not during the sham‐taVNS trial. Thus, it is reasonable to conclude that taVNS shifted the autonomic balance towards parasympathetic dominance. Since absolute low‐frequency and high‐frequency heart rate variabilities as well as SDNN and RMSSD were not significantly different between the taVNS and sham‐taVNS trials, we cannot answer the question whether the shift in autonomic balance was due to increased parasympathetic or decreased sympathetic tone or a combination of both. However, one may argue that the lack of an effect of taVNS on RMSSD and absolute high‐frequency heart rate variability suggest that taVNS reduced the sympathetic response to the MAST which may have contributed to the suppression of the HPA axis in the taVNS trial.

Based on the physiological time course of cortisol levels, we expected a continuous decline in salivary cortisol levels throughout the 100 min of the experimental protocol (from B1 to R4, see Figure 1). However, during the sham‐taVNS trial absolute salivary cortisol levels remained relatively constant for the first 55 minutes of the protocol (time points B1 to VS in Figure 3 top left) that included the MAST. This deviation from the physiologically expected time course suggests that the MAST triggered cortisol release that opposed the physiological decline in cortisol levels throughout the time course of the protocol. In contrast to the sham‐taVNS trial, absolute cortisol levels continuously declined throughout the first 55 min of the protocol during the taVNS trial despite the application of the MAST. As a result, salivary cortisol levels during the MAST were significantly lower during the taVNS protocol than during the sham‐taVNS protocol when cortisol levels were expressed as percent from baseline (time point VS in Figure 3 top right). Because of the physiological time course of cortisol levels, we conducted subgroup analyses for the experiments conducted in the mornings and afternoons (Figure 4, bottom). As expected, the inhibitory effect of taVNS on cortisol release in response to the MAST was confirmed in the morning experiments but was less pronounced during the afternoon. Collectively, these results are consistent with the notion that taVNS inhibits the cortisol response to stress.

There is strong evidence that afferent vagal nerve signaling constitutes the major sensory pathway of the anti‐inflammatory reflex (Tracey, 2002) through which information on inflammatory insults in the periphery are conveyed to the central nervous system and particularly to the nucleus of the solitary tract (NTS) (Berthoud & Neuhuber, 2000). Importantly, this afferent vagal pathway can also be activated through electrical stimulation of the auricular branch of the vagus nerve that innervates the cymba conchae of the ear (Peuker & Filler, 2002). Just as the sensory pathway of the anti‐inflammatory reflex, the auricular branch of the vagus nerve has been demonstrated to project to the NTS (Nomura & Mizuno, 1984; Toschi et al., 2023; Yakunina et al., 2017). Thus, it is reasonable to believe that taVNS activates central nervous system targets that are part of the anti‐inflammatory reflex. While autonomic innervation of the spleen is an established pathway of the efferent arc of the anti‐inflammatory reflex (Martelli et al., 2014; Olofsson et al., 2012), the role of the HPA axis has been less explored. Because the inflammatory reflex is anti‐inflammatory in nature, it has been generally assumed that activation of this reflex activates the HPA axis, resulting in adrenal cortisol release that would then contribute to the anti‐inflammatory effects of the inflammatory reflex (Bonaz et al., 2016; Fang et al., 2023). However, direct evidence for this assumption is scant. Warren et al. (2019) and D'Agostini et al. (2021) reported that taVNS prevented the diurnal decrease in salivary cortisol levels in novelty processing and learning paradigms, respectively. However, these studies did not focus on chronic inflammation or stress. Contrary to the general belief, our results appear to demonstrate that taVNS may actually inhibit stress‐induced activation of the HPA axis.

The “Glucocorticoid‐Resistance Model” proposed by Miller, Cohen, and Ritchey (Miller et al., 2002) over 20 years ago offers an alternative view on the role of the HPA axis in chronic inflammatory conditions. Based on this model, chronic stress impairs the immune system's capacity to respond to hormonal signals, such as glucocorticoids, that normally terminate inflammation. In line with this reasoning, glucocorticoid resistance has been implicated with inflammatory conditions, including asthma (Allam et al., 2023; Enweasor et al., 2021), chronic obstructive pulmonary disease (Zhou et al., 2023), and chronic rhinosinusitis (Chen et al., 2023). Furthermore, a systematic review across mouse, primate, and human studies found that chronic stress is associated with downregulation of cellular glucocorticoid receptors and upregulation of pro‐inflammatory biomarkers (Walsh et al., 2021). In addition to glucocorticoid resistance, a dysfunctional HPA axis with reduced adrenal responsiveness to pituitary signals may also contribute to inflammatory conditions. For example, it has been demonstrated that psoriasis patients with high stress scores have high adrenocorticotrophic hormone (ACTH) but low cortisol levels (Rajasekharan et al., 2023). In addition, the ACTH to cortisol ratio measured in the morning has been demonstrated to correlate positively with disease severity (Pietrzak et al., 2020). Likewise, cortisol levels have repeatedly been demonstrated to be lower in psoriasis patients than healthy controls despite higher self‐reported stress levels in psoriasis patients (Gisondi et al., 2021; Repousi et al., 2022). Thus, in addition to glucocorticoid resistance, patients with chronic inflammatory conditions may also develop resistance of the adrenal gland to properly respond to ACTH. In this study, we report that chronic taVNS for 3 months lowered diurnal cortisol levels in a psoriasis patient, a finding that is consistent with our data showing that taVNS inhibited the salivary cortisol response to a MAST. If chronic taVNS can restore HPA‐axis dysfunction and, therefore, improve chronic inflammatory conditions, such as psoriasis remains to be investigated. It is possible that taVNS through its afferent vagal signaling to the NTS activates inhibitory pathways projecting to the hypothalamic PVN, resulting in reduced corticotropin‐releasing hormone (CRH) secretion into the pituitary gland. Such a mechanism would restore the chronically elevated ACTH levels that have been reported in psoriasis patients (Rajasekharan et al., 2023), improve the ACTH to cortisol ratio (Pietrzak et al., 2020), and potentially reduce disease severity. In fact, preliminary unpublished data from our ongoing psoriasis study demonstrate that chronic taVNS improves disease severity in patients with plaque psoriasis.

The “hunger hormone” ghrelin modulates neuronal activity in the PVN (Nakazato et al., 2001) and may provide a mechanistic link between obesity and chronic inflammation. Ghrelin has been demonstrated to increase c‐FOS expression in the PVN (Cabral et al., 2016; Thomas et al., 2016), and electrophysiological experiments in rats demonstrated that ghrelin activates glucose‐responding neurons in the parvocellular part of the PVN (Chen et al., 2005). Furthermore, ghrelin administration in humans increases plasma levels of ACTH and cortisol (Coiro et al., 2005, 2011). Thus, there is strong evidence that ghrelin activates the HPA axis (Dos‐Santos et al., 2019). Importantly, in a previous study, we demonstrated that taVNS inhibits ghrelin (Kozorosky et al., 2022). Thus, it would be interesting to explore the possibility that inhibition of ghrelin may be mechanistically linked to the suppression of the HPA axis in response to taVNS.

There are some limitations with our study that need to be considered in the interpretation of our results. First, some experiments were conducted in the morning (*n* = 7) and some in the afternoon (*n* = 5). However, each of the twelve subjects reported for both trials (the taVNS and sham‐taVNS session) at the same time of the day. When designing the study, we expected that morning cortisol levels would be higher than afternoon levels, which would better unmask an inhibitory effect of taVNS on the HPA axis. On the other hand, the MAST would be more effective to raise cortisol levels in the afternoon and, therefore, better demonstrate a potential inhibition of the cortisol response to the MAST by taVNS. Based on these considerations, we did not restrict the experiments to a specific time of the day. It turned out that baseline cortisol levels were not different in the morning and afternoon experiments (Figure 4, bottom row). Thus, it is likely that being part of the study caused some activation of the HPA axis that negated the circadian effects on cortisol levels. Second, we did not measure CRH and ACTH levels. Thus, we cannot locate the site at which taVNS inhibits the HPA axis. Potential sites are the parvocellular part of the PVN, the anterior lobe of the pituitary gland, and the zona fasciculata of the adrenal cortex. Afferent nerve fibers of the auricular branch of the vagus nerve that are activated by taVNS project to the NTS (Nomura & Mizuno, 1984; Toschi et al., 2023; Yakunina et al., 2017). Neuronal tracing (Ben Musa et al., 2024) and optogenetic (Wang et al., 2019) experiments have identified pathways between the NTS and CRH‐positive neurons within the PVN. Furthermore, viral tracing studies identified direct pathways between the NTS and inhibitory GABA‐ergic neurons surrounding the PVN (Affleck et al., 2012). Based on these neuroanatomical pathways, it is reasonable to assume that taVNS inhibits the HPA axis at the level of the PVN. However, because CRH was not measured in our experiments, this assumption remains speculative. Finally, the chronic inhibition of the HPA axis by daily taVNS for up to 3 months seen in the case study of the psoriasis patient is consistent with the acute inhibition of the cortisol response to the MAST. However, this finding is based on an observation in a single patient and, therefore, cannot be generalized. Furthermore, this psoriasis patient had very mild psoriasis with a Psoriasis Area and Severity Index of only 1.5 (0–5 is considered mild, 6–10 is considered moderate, and >11 is considered severe). Thus, it remains unclear if taVNS would also inhibit cortisol in patients with more severe psoriasis.

In conclusion, this study demonstrates that taVNS acutely inhibits the salivary cortisol response to a MAST. Even though the presented patient case study suggests that taVNS also suppresses the HPA axis chronically, this incidental finding needs to be confirmed by properly powered studies. Collectively, our study, together with the mounting evidence of HPA‐axis dysregulation in chronic inflammatory diseases provides a rationale for future studies exploring the exact mechanisms through which vagus nerve stimulation may affect the HPA axis and investigating if afferent VNS through non‐invasive taVNS can restore HPA‐axis function in chronic inflammatory diseases and thereby improve clinical disease severity.

## FUNDING INFORMATION

This study was supported by funds from the Office of Research and Sponsored Programs at Burrell College of Osteopathic Medicine, a grant from the American Osteopathic Association (Grant No.: 19137759), and a Summer Student Research Grant provided to AMcL by the National Psoriasis Foundation.

## CONFLICT OF INTEREST STATEMENT

The authors have no conflict of interest to declare.

## ETHICS STATEMENT

The study was approved by the Burrell College Institutional Review Board (BURRELL IRB 0112_2023) and all participants provided informed written consent.

## Data Availability

The data supporting the findings of this study are available from the corresponding author, upon reasonable request and upon approval of data sharing by the Burrell College Institutional Review Board.
